# Disruptive natural selection predicts divergence between the sexes during adaptive radiation

**DOI:** 10.1002/ece3.2868

**Published:** 2017-04-11

**Authors:** Stephen P. De Lisle, Locke Rowe

**Affiliations:** ^1^ Department of Ecology and Evolutionary BiologyUniversity of TorontoTorontoONCanada

**Keywords:** adaptive radiation, character displacement, disruptive selection, *Notophthalmus*, sexual dimorphism

## Abstract

Evolution of sexual dimorphism in ecologically relevant traits, for example, via resource competition between the sexes, is traditionally envisioned to stall the progress of adaptive radiation. An alternative view is that evolution of ecological sexual dimorphism could in fact play an important positive role by facilitating sex‐specific adaptation. How competition‐driven disruptive selection, ecological sexual dimorphism, and speciation interact during real adaptive radiations is thus a critical and open empirical question. Here, we examine the relationships between these three processes in a clade of salamanders that has recently radiated into divergent niches associated with an aquatic life cycle. We find that morphological divergence between the sexes has occurred in a combination of head shape traits that are under disruptive natural selection within breeding ponds, while divergence among species means has occurred independently of this disruptive selection. Further, we find that adaptation to aquatic life is associated with increased sexual dimorphism across taxa, consistent with the hypothesis of clade‐wide character displacement between the sexes. Our results suggest the evolution of ecological sexual dimorphism may play a key role in niche divergence among nascent species and demonstrate that ecological sexual dimorphism and ecological speciation can and do evolve concurrently in the early stages of adaptive radiation.

## 1 Introduction

The ecological theory of adaptive radiation suggests lineages diverge to exploit ecological opportunity, appealing to evolutionary biologists by simultaneously explaining diversity of species, their phenotypes, and the habitats in which both reside (Schluter, 2000). Yet for organisms with separate sexes, striking phenotypic divergence between males and females of the same species implies that a large component of adaptation is achieved via the evolution of sexual dimorphism (Darwin, 1871; Fairbairn, 2013). The role of this within‐species divergence in adaptive radiation becomes especially interesting in the case of sexual dimorphism in ecologically relevant traits, such as body size and feeding morphology, because theory suggests that these ecological sexual dimorphisms can be driven by the same processes of resource competition and niche divergence that are envisioned to be key drivers of ecological speciation during adaptive radiations of genetically independent lineages (Slatkin, 1984). The idea that the same ecological forces may underlie the evolution of ecological sexual dimorphism and phenotypic divergence between nascent species premises a long‐held supposition that the evolution of ecological sexual dimorphism may thus stall the progress of speciation and adaptive radiation. This idea has been formalized for the case of speciation under sympatry (Bolnick & Doebeli, 2003; Cooper, Gilman, & Boughman, 2011; Van Dooren, Durinx, & Demon, 2004), and verbally generalized to adaptive radiation in general (Butler, Sawyer, & Losos, 2007). Theory predicts the dimorphism‐as‐constraint hypothesis to manifest as a correspondence between divergent selection and phenotypic divergence between the sexes and species over the course of diversification. This prediction would be expected even (or perhaps especially) if speciation has not occurred in sympatry; unless genetic constraints on sexual dimorphism are complete, sexual dimorphism is expected to evolve before sympatric speciation (Bolnick & Doebeli, 2003) in these models and thus the constraint hypothesis may be especially relevant in clades characterized by allopatric speciation.

An alternative view is that the evolution of ecological sexual dimorphism may often play an important *positive* role in adaptive radiation (De Lisle & Rowe, 2015b). For example, under the likely common conditions of allopatric divergence between nascent species (Coyne & Orr, 2004), and sex‐specific phenotypic optima (Cox & Calsbeek, 2009), Lande's (1980) model of the evolution of sexual dimorphism leads to the prediction that population mean fitness (and thus probability of establishment of a nascent allopatric species) depends on the ability of males and females to reach their phenotypic optima. This suggests that rapid evolution of sexual dimorphism will play an important role in successful establishment of a nascent allopatric species whenever phenotypic optima differ for the sexes. In the case of sexually antagonistic (SA) natural selection driven by frequency‐dependent resource competition (Slatkin, 1984), the predicted positive effect of dimorphism on species establishment may even be exacerbated as the strength of intraspecific resource competition is reduced during the course of ecological character displacement between the sexes. Although a direct test of this extension of Lande's model is difficult, one signature would be the evolution of ecological sexual dimorphism that increases local adaptation during niche divergence associated with ecological speciation.

Direct empirical tests of the role of ecological sexual dimorphism in adaptive radiation are limited. Past studies have attempted to relate patterns of macroevolutionary diversification and community assembly to measures of sexual dimorphism (Butler et al., 2007; Dayan & Simberloff, 1994; De Lisle & Rowe, 2015b; Hendry, Guiher, & Pyron, 2014; Schoener, 1977; Stephens & Wiens, 2009). Although these studies are interesting, they are somewhat unsatisfying in that (1) all predictions for a role of ecological sexual dimorphism in adaptive radiation depend upon processes unfolding in the early stages of ecological speciation and (2) these past studies have no direct evidence of an ecological cause of sexual dimorphism.

Here, we examine the joint evolution of phenotypic divergence between the sexes and among lineages in a relatively young clade of North American salamanders, the newts *Notophthalmus*. All three species of *Notophthalmus* are generalist semiaquatic predators that breed in ponds and lakes (Petranka, 1998). The most well studied species, the eastern newt *N. viridescens* has diverged rapidly as the last ice age into four subspecies adapted to unique niches associated with aquatic life (Takahashi & Parris, 2008; Takahashi et al., 2014). *N. v. viridescens* inhabits Appalachian upland forest and temporary ponds and is adapted to spend much of its life on land, with a terrestrial juvenile stage and adult migration out of the aquatic environment outside of the breeding season (Sever, 2006). Alternatively, the nearly fully aquatic *N. v. piaropicola* inhabits permanent water bodies in peninsular Florida, skipping terrestrial phases and rarely leaving water; *N*. *v. dorsalis* and *N. v. louisianensis* reflect intermediate life histories, plastically skipping the terrestrial eft phase and maturing directly into aquatic adults under favorable conditions (Croshaw et al., 2013; Petranka, 1998; Sever, 2006; Takahashi & Parris, 2008; Takahashi, Takahashi, & Parris, 2011; Takahashi et al., 2014). These differences in aquatic life cycle reflect adaptation to differences in both the quality of terrestrial habitat and breeding pond permanence (Sever, 2006; Takahashi & Parris, 2008). The natural histories of *N. meridionalis* and *N. perstriatus*, the two other species in the genus, have been characterized in much less detail although evidence suggests *N. perstriatus* may be adapted for predominately aquatic life (Petranka, 1998).

**Figure 1:**
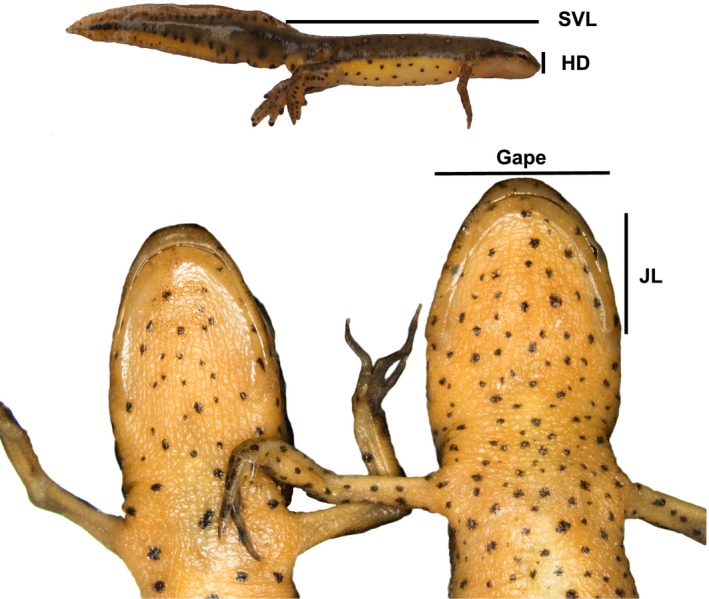
Sexual dimorphism in head shape in *N. viridescens* (ventral view). Males (left) have narrower gapes and longer lower jaws than females (right), which tend to have shorter jaws for their size and wider gapes. A representative lateral view is provided at the top. The four linear traits measured are indicated by black lines. SVL, snout‐vent length; HD, head depth; JL, jaw length

Past work shows a significant ecological component to sexual dimorphism in *N. viridescens,* in particular *N. v. viridescens*. Males and females have diverged subtly but significantly in head shape; females have wider gapes and shorter, shallower heads for their size than do males (Figure 1), and this morphological dimorphism is associated with divergence in diet (De Lisle & Rowe, 2015a). The sexes also exhibit divergent within‐pond microhabitat use, parasite loads, and sensitivity to heterospecific resource competitors (Grayson, De Lisle, Jackson, Black, & Crespi, 2012; De Lisle & Rowe, 2014; De Lisle & Rowe, 2015a, 2015c). Using a series of artificial pond experiments, we have shown that this ecological sexual dimorphism is in part an outcome of resource competition‐driven disruptive natural selection (De Lisle & Rowe, 2015a). Fitness and selection within ponds is negative frequency (sex ratio)‐dependent, and the strength of competition mediates the strength of disruptive, SA natural selection on morphology. These results are consistent with models of disruptive selection driven by frequency‐dependent resource competition and provide one of the most explicit tests of ecological character displacement between the sexes. Thus, past work suggests that morphological sexual dimorphism in *N. v. viridescens* is at least in part an outcome of SA natural selection, with other sources of SA selection more directly related to the sex roles also likely playing a role (De Lisle & Rowe, 2015a).

Our finding of disruptive natural selection within breeding ponds in *N. v. viridescens*, combined with recent adaptive ecological divergence among subspecies and species of *Notophthalmus*, allows for an empirical test of competition's potential role in both the evolution of sexual dimorphism and adaptation during speciation in a young adaptive radiation. Specifically, we can make three predictions: if similar processes of resource competition drive evolution of sexual dimorphism across the genus, then (1) we expect SA natural selection to align with morphological divergence between the sexes, and (2) the extent of sexual dimorphism to associate with an aquatic life history. This prediction can be made on the basis of biomechanical trade‐offs between foraging in aquatic (via bidirectional suction) versus terrestrial (via tongue prohesion) habitat (Deban & Wake, 2000; Wake & Deban, 2000); there is an a priori expectation that selection on multivariate feeding morphology would differ fundamentally across these habitats, as reflected in the intermediate feeding morphology that characterizes semiaquatic newts (Deban & Wake, 2000; Heiss, Aerts, & Van Wassenbergh, 2013) and the morphological changes that underlie transitions between these habitats in salamanders and other vertebrates (Schwenk, 2000). Thus, if character displacement between the sexes prevails in aquatic habitat then we expect correlated evolution of sexual dimorphism and adaptation to aquatic life. Finally, (3) if the evolution of sexual dimorphism constrains diversification, then we would expect a correspondence between disruptive natural selection, phenotypic divergence between the sexes, and divergence between species. Note that this prediction holds regardless of how speciation has proceeded.

## 2 Materials and methods

### 2.1 Theoretical background: geometry of sexual dimorphism

Sexual dimorphism can be described geometrically by a canonical discriminant analysis (De Lisle & Rowe, 2015a), where the direction through trait space that defines the maximum amount of divergence (relative to the phenotypic variance) between the sexes is described by the vector of canonical coefficients

(1) $$c=P^{-1}\left({\overset{¯}{z}}_{m}-\;{\overset{¯}{z}}_{f}\right)$$

where **P** is the pooled phenotypic covariance matrix and $\overset{¯}{z}$ is a column vector of sex‐specific phenotypic means (Campbell & Atchley, 1981; Mitteroecker & Bookstein, 2011). The canonical coefficient vector **c** is related to the magnitude of multivariate sexual dimorphism by

(2) $${\left({\overset{¯}{z}}_{m}-{\overset{¯}{z}}_{f}\right)}^{T}c=D^{2}$$

where *D* ^2^ is the Mahalanobis distance between the sexes. Thus, the vector **c** is a measure of variance‐standardized multivariate sexual dimorphism that captures an element of both the orientation and magnitude of divergence between the sexes that is comparable across populations and taxa (*n.b*. if the data are standardized to unit variance prior to calculating **c** (the total sample standardized canonical coefficients), such a comparison is mathematically equivalent and conceptually related to among‐group comparisons of standardized linear selection gradients β , as β can be defined as the vector of canonical coefficients defining the population before and after selection (Mitteroecker & Bookstein, 2011)).

Evolutionary change in variance‐standardized sexual dimorphism can then be described as

(3) $$\Delta c\;=P^{-1\prime }\left({\overset{¯}{z}}_{m}^{\prime }-\;{\overset{¯}{z}}_{f}^{\prime }\right)-P^{-1}\left({\overset{¯}{z}}_{m}-\;{\overset{¯}{z}}_{f}\right)$$

where the primes denote parameters occurring in some set after a selective epidose(s). In a group of multiple independently evolving populations Equation (3) would extend to a variance and total evolutionary divergence in multivariate sexual dimorphism across a clade can be described by the second‐order tensor

(4) $$S\;\;=\;\;\left[\begin{array}{ccc} \sigma^{2}\left(c_{1}\right) & \cdots & \sigma \left(c_{1},\;c_{k}\right)\\ \vdots & \ddots & \vdots \\ \sigma \left(c_{k},\;c_{1}\right) & \cdots & \sigma^{2}\left(c_{k}\right) \end{array}\right]$$

where the diagonals of **S** describe the among‐taxa variance in the standardized canonical coefficients (*c*_1*−k*_, where *k* is the number of traits in the canonical discriminant analysis) and the off‐diagonals describe the among‐taxa covariance between standardized coefficients. Thus, **S** is a covariance matrix whose first eigenvector, **s**_**max**_, describes the direction through trait space where species have diverged the most in multivariate sexual dimorphism (for a discussion of the use of higher order tensors in comparative quantitative genetic analyses, see Hine, Chenoweth, Rundle, & Blows, 2009; Aquirre, Hine, McGuigan, & Blows, 2014).

Importantly, Equations (1) and (3) imply that when selection is SA, one prediction is that sexual dimorphism may evolve so that $\Delta \left({\overset{¯}{z}}_{m}-\;{\overset{¯}{z}}_{f}\right)$ and thus Δc and $c^{\prime }=P^{-1}\left({\overset{¯}{z}}_{m}^{\prime }-{\overset{¯}{z}}_{f}^{\prime }\right)$ aligns with the direction of maximum SA selection (see, e.g., Lande, 1980; Wyman, Stinchcombe, & Rowe, 2013). In the case of competition‐driven frequency‐dependent disruptive natural selection (i.e., ecological character displacement between the sexes), the first eigenvector of the **γ** matrix of nonlinear selection is the theoretically appropriate description of the direction of maximum selection (De Lisle & Rowe, 2015a). If the same pattern of SA selection is conserved through the history of a radiation, as, for example, could be the case under phenotype‐mediated frequency‐dependent resource competition (Rueffler, Van Dooren, Leimar, & Abrams, 2006), then among‐lineage lineage variation in **c** captured by the first eigenvector of **S** may be predicted to align with this pervasive SA selection. Alternatively, if speciation has occurred under sympatry and was driven by competition‐driven disruptive selection, then conserved disruptive selection may instead be expected to align with among‐species variance in phenotypic grand means, **D**. Geometric comparisons of variation in dimorphism **S**, selection, and total among‐species phenotypic divergence **D** then allow for tests of how, and perhaps even why, the sexes and species diverge during adaptive radiation.

### 2.2 Data collection

**Figure 2:**
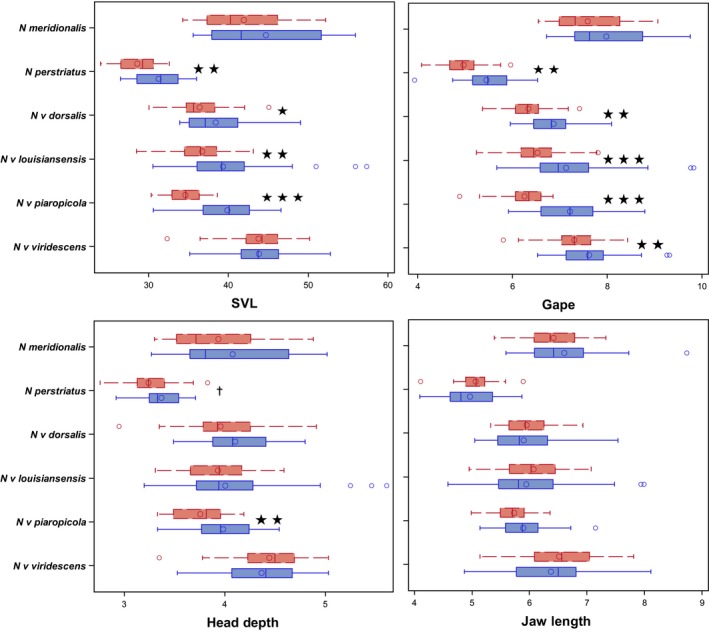
Distributions of raw trait values for males (red‐dashed) and females (blue‐solid) across *Notophthalmus*. Stars indicate statistical significance at alpha* *= 0.05, 0.01, 0.0001 (one, two, three, respectively). Cross indicates *p *< .10. Units are millimeters. SVL, snout‐vent length

**Figure 3:**
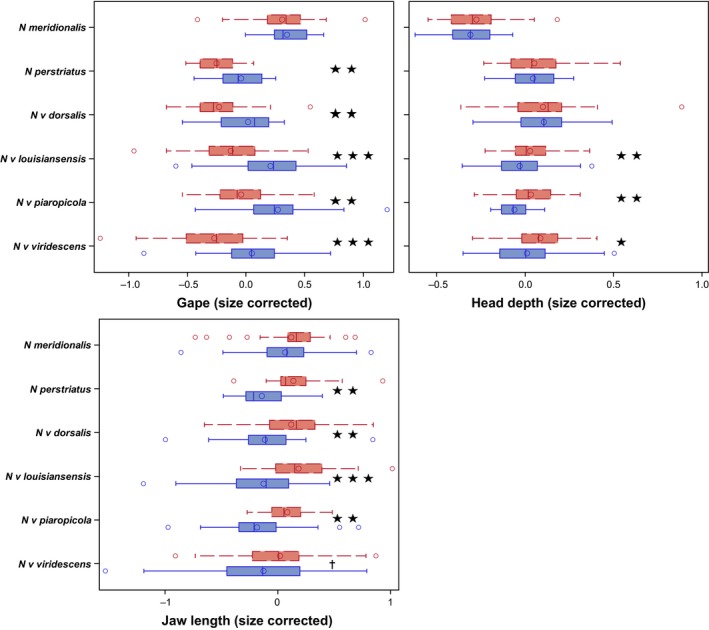
Distributions of size‐corrected head trait values for males (red‐dashed) and females (blue‐solid) across *Notophthalmus*. Values are residuals from linear regressions of gape, head depth, and jaw length on the first principle component of the total phenotypic covariance matrix of the full dataset (i.e., including body length SVL). Stars indicate statistical significance at alpha* *= 0.05, 0.01, 0.0001 (one, two, three, respectively). Cross indicates *p *< .10

We examined and measured specimens of *Notophthalmus* from the Carnegie Museum of Natural History, the Smithsonian Museum of Natural History, and the American Museum of Natural History. Subspecies of *N. viridescens* were identified by locality and coloration (i.e., dorsal spot/stripe pattern Petranka, 1998). We avoided measuring individuals from phenotypically intergraded populations at the subspecies range margins because assigning subspecies status to such individuals/populations is tenuous. We sexed adult specimens based on cloacal morphology and secondary sex traits when present (e.g., nuptial pads) and confirmed sex by examination of gonads for any specimens whose abdomens had been previously incised. For each specimen, we measured snout‐vent length, gape, lower jaw length, and head depth (from the lower right jaw to the top of the orbital skeletal ridge) to two decimal places using digital calipers (Figure 1). These morphological traits are independent of the characters used to assign sex, and are the same traits measured in the same way as in our past work on character displacement in *N. v. viridescens* (De Lisle & Rowe, 2015a). Our sampling was limited by rareness of some species and subspecies, and we excluded measurements from 20 neotene paedomorphs from one population of *N. perstriatus*; these individuals had fully developed gonads yet retained a larval body plan, and were thus phenotypically distinct from other individuals of *N. perstriatus* (multivariate mixed model: life history effect, *F*_1, 63_ = 5.06, *p *= .028; life history*trait *F*_3,61_ **= 17.69, *p *< .0001). In total, our final dataset consisted of measurements from 477 specimens from males and females of all species and subspecies in the genus, although we were unable to obtain enough data for separate analysis of the two subspecies of *N. meridionalis*. Summary statistics of all measurements taken, and sample sizes are given in Table S1. Sex ratios were not significantly different from 50:50 for any taxon (all *p *> .09) or the pooled data (*p *= .49). Distributions for each trait*sex*taxon combination are summarized in Figure 2. We also present size corrected (residuals from a regression of trait values on the first principal component of the total phenotypic correlation matrix; an approach that is conceptually analogous to our multivariate analyses) trait distributions to illustrate trait variation independent of the major axis of phenotypic variance in Figure 3.

### 2.3 Statistical analysis

Our analysis entailed two general approaches. First, we fit multivariate and univariate mixed models with fixed effects of taxa, sex, and their interactions to test the hypotheses that sexual dimorphism and among‐species differences are statistically significant. We then fit a second series of mixed models to estimate **S** and **D** matrices, which were used for geometric comparisons with selection that are most consistent with theory (e.g., see above; Theoretical background section). This approach allows us to both leverage well‐developed and powerful fixed‐effect hypothesis tests in addition to exploring the geometry of among‐taxa variation. Note it is not feasible to estimate both the hypothesis tests of fixed effects and the random effect covariance matrices in a common model; for example, an among‐species phenotypic covariance matrix **D** would be conditioned on any fixed effect of sex, making geometric comparisons with sexual dimorphism a trivial outcome of the model specification.

To assess whether sexual dimorphism exists across the genus and differed across taxa, we fit a multivariate mixed model with trait values as the response vector, and trait type, sex, taxon (*N. perstriatus, N. meridionalis, N. v. ssp*), and all two‐ and three‐way interactions as fixed effects. We included trait as an R‐side (repeated measures) random effect with individual as the subject, modelling a separate phenotypic covariance matrix for each taxon. This residual covariance structure fit better than a common phenotypic covariance (∆AIC* *= 59.66). To compare trait‐specific effects, we also present univariate mixed‐model analyses. These models included sex, taxon, and their interaction as fixed effects, and the taxon‐specific residual variance as a random effect. We performed univariate tests on both the raw trait values and size‐corrected head traits (residuals from a least‐squares regression of gape, jaw length, or head depth on the first principle component of the total phenotypic correlation matrix for all four traits) to illustrate patterns in the data that are independent of size; note that multivariate analyses can accommodate such size‐independent effects because the covariance structure of the data is explicitly modeled.

We repeated our multivariate analysis, but limited to subspecies of *N. viridescens*, to test the hypothesis that dimorphism differs across subspecies of *N. viridescens*. We quantified the extent of sexual dimorphism for visual presentation by Mahalanobis distance between the sexes, which is a standardized estimate of multivariate effect size. Confidence intervals for Mahalanobis distance were obtained from percentiles of the sampling distribution constructed from a nonparametric bootstrap (1,000×).

We estimated the among taxa divergence matrix, **D** (i.e., the among‐species/subspecies phenotypic covariance matrix (Lande, 1979)), by fitting a multivariate mixed model with trait values (both sexes pooled in the same analysis) as the response vector and and trait type as a fixed effect to center the data. This model included a G‐side random effect of trait type with taxon as the subject to estimate **D**. We accommodated within‐taxa (co)variances by also modelling an R‐side random effect of trait type with individual nested within taxon as the subject. For this analysis, we standardized traits globally (across all taxa, with data for males and females pooled) to zero mean and unit variance, in order to make meaningful comparison with estimates of variance‐standardized within‐taxa nonlinear selection, where within‐population data were standardized in the same way (see below). To estimate **S**, we fit a multivariate mixed model with the vector of total sample standardized canonical coefficients (estimated and standardized separately for each taxon) as the response and trait type as a fixed effect to center the data. We fit trait type as a G‐side random effect with taxon as the subject to estimate **S**. To accommodate uncertainty in our estimates of the canonical coefficients, we analyzed bootstrapped data, where we resampled (100x) our data and each time estimated **c** for each taxon, and then analyzed the set of bootstrapped **c** estimates treating bootstrap replicate as an R‐side (repeated measures) random effect with replicate nested within taxa as the subject. Although in theory it would be possible to fit one mixed model where **D** is estimated as the covariance in random intercepts among taxa, while **S** is the covariance in the sex effect among taxa, fitting this model entails either estimating excessive (and likely meaningless) parameters (i.e., where the intercept and sex*trait effect are included in the same random term) or fitting two random terms to the same set of subjects, which failed to converge.

Our approach to data standardization aimed to be as consistent as possible across analyses; in no case did we standardize separately for males and females, as this was not performed in past work (De Lisle & Rowe, 2015a) and would result in nonlinear discriminant analyses. None the less, we explored a variety of standardizations and conclusions remain unchanged in all cases. We also obtained the same results using the mean discriminant vector (rather than **s**_**max**_) to describe divergence between the sexes.

We calculated the association between divergence in sexual dimorphism, **s**_**max**_, or divergence among species, quantified as **d**_**max**_, the first eigenvector of **D**, and our within‐taxon estimate of disruptive selection as the vector correlations (projections) **s**_**max**_^**T**^ **γ**_**max**_ and **d**_**max**_^**T**^ **γ**_**max**_, where T is matrix transpose and **γ**_**max**_ is the first eigenvector of the **γ** matrix of nonlinear standardized selection gradients from the high density experiment in (De Lisle & Rowe, 2015a), where the strength of competition was strongest. We obtained qualitatively equivalent conclusions using the average of all three estimates of **γ** from (De Lisle & Rowe, 2015a). Briefly, we estimated **γ** from artificial‐pond experiments with relative growth rate as a fitness measure, with both sexes pooled in the same mixed model multiple quadratic regression. Traits were standardized to zero mean and unit variance across the experiment. Significance of dominant eigenvalues was determined by a randomization test. Details of these experiments and analyses are provided in (De Lisle & Rowe, 2015a). We empirically constructed sampling distributions for the correlations between disruptive selection and **s**_**max**_ and **d**_**max**_ by resampling from a multivariate normal distribution with mean vector equal to the original REML estimates of **S** or **D** and covariance proportional to the inverse of the Hessian matrix of the optimized likelihood from the mixed model used to estimate **S** or **D**. For each of 100,000 samples we recomputed **s**_**max**_ and **d**_**max**_, the vector correlation of each with **γ**_**max**_ **,** and the vector correlation between **s**_**max**_ and **d**_**max**_. This approach of resampling the information matrix is a robust method for constructing confidence intervals for arbitrary linear or nonlinear functions of random‐effect covariance parameters estimated from mixed models (Houle & Meyer, 2013, 2015), and has a major computational advantage over bootstrapping because the mixed model is only fit once. Our comparisons of **S** and **D** with **γ** were necessarily limited to the first eigenvector because this was the only statistically significant dimension of nonlinear selection (De Lisle & Rowe, 2015a) and a vast majority of the variance in **D** (96%) and **S** (88%) was captured in one dimension.

In some cases, appropriate tests of hypotheses that relate microevolutionary parameters to among‐taxa, macroevolutionary divergence require the incorporation of quantitative information on phylogenetic relatedness. For example, tests of evolution by drift, along genetic lines of least resistance, or evolution toward an optimum phenotype are most appropriately performed by parameterizing a family of Brownian motion process models of evolution (Hansen, 1997; Hohenlohe & Arnold, 2008). Although Brownian motion rates are often interpreted in light of selection, we have no well‐developed macroevolutionary process models that incorporate frequency‐dependent disruptive selection, a phylogeny, and are parameterized to relate to microevolutionary processes; such a model would need to accommodate the likely possibility that this selection shapes both phenotypic evolution and the branching pattern on the tree. Further, existing OU models that incorporate (stabilizing) nonlinear selection cannot be distinguished from simple multivariate Brownian motion with residual variance (De Lisle, unpublished simulations). Thus, in our case, a phylogenetically agnostic analysis is appropriate and we are really most interested in how taxon means have diverged (for a related discussion, see Kelly & Price, 2004). Nonetheless, we present an analysis of phylogenetically informed, Brownian motion analogs of **D** and **S** in the Appendix S1 for completeness. Conclusions on the relationship with **γ**_**max**_ remained qualitatively equivalent and quantitatively similar.

All statistical analyses were performed in SAS/IML 9.3 (SAS institute, Cary, NC, USA), with the exception of supplementary analysis performed in R. Mixed models were fit by REML in the glimmix procedure. All random effect covariance matrices were modeled as the Cholesky parameterization of an unstructured covariance matrix to insure positive semidefiniteness. We used the Kenwood–Roger approximation for degrees of freedom for models with fixed effects, and report type III Wald *F* tests for these effects (Littell, Milliken, Stroup, Wolfinger, & Schabenberger, 2006).

## 3 Results

We found significant multivariate sexual dimorphism in morphology across the genus *Notophthalmus* (multivariate mixed model: sex effect, *F*_1, 133.7_ **= 31.89, *p *< .0001), and sexual dimorphism differed significantly across species and subspecies (multivariate mixed model: sex*taxon effect, *F*_5, 150.4_ **= 4.18, *p *= .0014; sex*taxon*trait *F*_15, 211.8_ **= 2.96, *p *= .0003). Univariate analyses reflect these results (Figures 2 and 3; Table 1), and also indicate sexual dimorphism primarily occurs in head shape, independent of body size, across the genus (*c.f*. Figure 2 [raw traits] and Figure 3 [size‐corrected traits]).

**Table 1:** Univariate analyses of morphological variation in *Notophthalmus*

Snout‐vent length					Gape				
Effect	Num. DF	Den. DF	*F*	*p*	Effect	Num. DF	Den. DF	*F*	*p*
Sex	1	129.3	33.78	<.0001	Sex	1	187.5	65.91	<.0001
Subsp	5	149.8	155.53	<.0001	Subsp	5	148.7	120.53	<.0001
Sex*subsp	5	149.8	4.69	.0005	Sex*subsp	5	148.7	2.22	.0553

Head depth					Jaw length				
Effect	Num. DF	Den. DF	*F*	*p*	Effect	Num. DF	Den. DF	*F*	*p*
Sex	1	155.4	6.85	.0097	Sex	1	221.1	0.05	.8227
Subsp	5	150	95.56	<.0001	Subsp	5	153.8	62.24	<.0001
Sex*subsp	5	150	2.08	.0712	Sex*subsp	5	153.8	1.29	.2699

Gape (size corrected)					Head depth (size corrected)				
Effect	Num. DF	Den. DF	*F*	*p*	Effect	Num. DF	Den. DF	*F*	*p*
Sex	1	267.7	89.73	<.0001	Sex	1	217.5	6.48	.0116
Subsp	5	153.1	28.47	<.0001	Subsp	5	145.3	30.47	<.0001
Sex*subsp	5	153.1	2.88	.0164	Sex*subsp	5	145.3	0.8	.5549

Jaw length (size corrected)									
Effect	Num. DF	Den. DF	*F*	*p*					
Sex	1	242.7	41.78	<.0001					
Subsp	5	148.3	1.56	.1749					
Sex*subsp	5	148.3	1.37	.2388					

**Figure 4:**
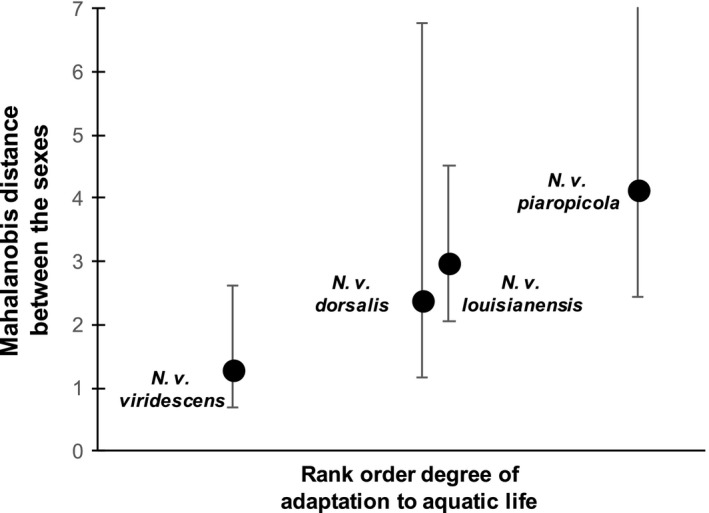
Magnitude of sexual dimorphism correlates with aquatic life history across *N. viridescens*. Ranks are based on natural history descriptions and experiments from the literature (see text). The Spearman correlation was statistically significant (Spearmen *r *= .95, *p *= .05, *df *= 2), as was the overall among subspecies difference in sexual dimorphism (multivariate LMM sex*subspecies *p *= .0002). Error bars are 95% bootstrapped confidence limits; the upper bound for *N. v. piaropicola* (10.33) was not included to save space. *N. v. dorsalis* and *N. v. louisianensis* were assigned the same rank

Across subspecies of *N. viridescens*, there is a rank order correlation between relative proportion of the life history spent in the aquatic phase and the extent of multivariate sexual dimorphism (Spearmen *r *= .95, *p *= .05, *df *= 2; Figure 4; dimorphism estimated as Mahalanobis distance between the sexes, *N. v. dorsalis* and *N. v. lousianensis* assigned the same rank (Takahashi & Parris, 2008)). These among‐subspecies differences in sexual dimorphism were statistically significant (multivariate mixed model: sex*subspecies *F*_3, 149.1_ **= 6.97, *p *= .0002, sex*subspecies*trait *F*_9, 188.4_ **= 3.39, *p *= .0007). Further, although too little is known about the natural histories of *N. meridionalis* and *N. perstriatus* to rank their life histories relative to *N. viridescens*, it is noteworthy that *N. perstriatus*, which has been reported to frequently remain aquatic for much of its life (Petranka, 1998), also exhibits strong sexual dimorphism (Mahalanobis distance* *= 3.54, 95% bootstrapped CI 2.17–8.95) and the small sample of fully paedomorphic *N. perstriatus* exhibited the strongest magnitude of sexual dimorphism (Mahalanobis distance* *= 7.33, 95% bootstrapped CI 4.66–35), although this difference in sexual dimorphism between forms of *N. perstriatus* was not statistically significant (multivariate mixed model: life history*sex effect, *F*_1,63_ **= .21, *p *= .65; life history*sex*trait *F*_3,61_ **= 0.86, *p *= .46).

**Figure 5:**
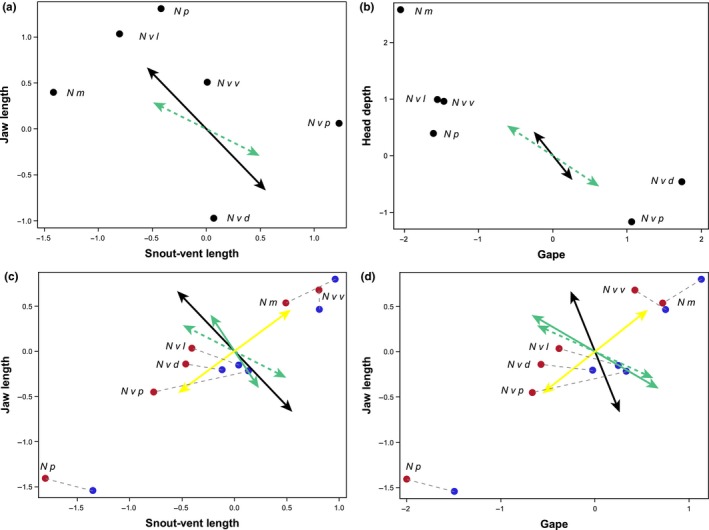
Divergence between the sexes correlates with within‐subspecies disruptive selection across *Notophthalmus*. The direction of maximum divergence between the sexes, **s**_**max**_ (green dashed arrows) defined as the first eigenvector of the covariance matrix **S** of canonical coefficients from taxon‐specific canonical discriminant analyses on the sexes, correlates strongly with the **γ**_**max**_ (black arrows), the direction of maximum disruptive selection measured in *N. v. viridescens*. The same qualitative conclusions were obtained with the mean vector of canonical coefficients (solid green arrows). In A and B, points are standardized canonical coefficients for each taxon, illustrating **S** in two dimensions. In C and D, points are taxon sex‐specific (red, male; blue, female) standardized mean trait values. Yellow arrow is divergence in taxon mean trait values, **d**_**max**_. Initials indicate species or subspecies

**Figure 6:**
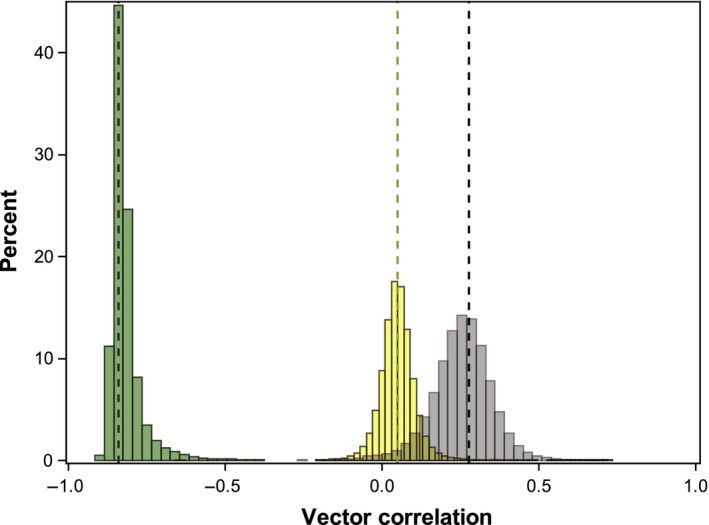
Sampling distributions for vector correlations between evolutionary parameters in *Notophthalmus*. The empirically constructed sampling distribution (see text) for the vector correlation between **s**_**max**_ (green) or **d**_**max**_ (yellow) and **γ**_**max**_, and the vector correlation between **s**_**max**_ and **d**_**max**_ (gray). Dashed lines are the original REML estimates

Phenotypic divergence among species and subspecies, as measured by the first eigenvector of the among taxa phenotypic covariance matrix, **d**_**max,**_ occurred nearly orthogonally to the within subspecies direction of maximum disruptive selection, **γ**_**max**_ (vector correlation *r*_*v*_ **= 0.0458; 95% confidence interval for *r*_*v*_ **= −0.064 to 0.176; Figures 5 and 6) and primarily reflects divergence in body size (Figure 5; high positive loadings for each trait in Table 2). Among‐taxa variation in sexual dimorphism, however, measured as the first eigenvector of the covariance matrix of canonical coefficients defining the sexes, **s**_**max,**_ was strongly aligned with **γ**_**max**_ (vector correlation *r*_*v*_ = −0.843; 95% confidence interval for *r*_*v*_ = −0.606 to −0.87; Figures 5 and 6). Nonoverlapping confidence intervals indicate that among‐species and among‐sex correlations with **γ**_**max**_ were also significantly different from each other, and the vector correlation between **s**_**max**_ and **d**_**max**_ indicate the association between total variation among species and variation in sexual dimorphism is weak (vector correlation *r*_*v*_ **= 0.276; 95% confidence interval for *r*_*v*_ **= 0.009–0.44; Figure 6). These geometric comparisons of covariance matrices support the patterns illustrated in Figures 2 and 3; variation among species primarily reflects variation in absolute trait size (Figure 2), while divergence between the sexes is most strongly associated with variation in head shape independent of body size (Figure 3).

**Table 2:** REML estimates and spectral decompositions of S and D matrices

Trait	SVL	Gape	Head Depth	Jaw Length	Eigen values	Eigenvectors			
						SVL	Gape	Head Depth	Jaw Length
**S**									
SVL	0.506	0.478	−0.502	−0.162	1.744	0.495	0.634	−0.531	−0.268
Gape	–	0.764	−0.544	−0.358	0.235	0.566	−0.513	−0.356	0.538
Head depth	–	–	0.525	0.203	0.009	−0.536	0.365	−0.393	0.652
Jaw length	–	–	–	0.198	0.005	0.384	0.450	0.661	0.462
**D**									
SVL	0.776	0.808	0.597	0.664	2.663	0.538	0.564	0.422	0.463
Gape	–	0.872	0.596	0.702	0.096	−0.071	−0.466	0.870	−0.142
Head depth	–	–	0.547	0.508	0.008	−0.785	0.571	0.241	−0.002
Jaw length	–	–	–	0.572	0.000	−0.298	−0.372	−0.081	0.875

## 4 Discussion

We have shown that morphological divergence between the sexes across a clade of salamanders has occurred in a combination of head‐shape traits that past work (De Lisle & Rowe, 2015a) has shown to be under disruptive, competition‐driven natural selection within breeding ponds. Morphological divergence between species and subspecies, however, has occurred orthogonally to this within‐pond disruptive selection and primarily reflects differences in body size. These data refute the hypothesis of ecological dimorphism‐as‐constraint, which predicts alignment between these three parameters. Across subspecies of the most phenotypically diverse species, *N. viridescens*, the magnitude of sexual dimorphism is highest in subspecies characterized by a predominantly aquatic life history, consistent with the hypothesis (De Lisle & Rowe, 2015a) that within‐pond disruptive selection is pervasive and contributes to ecological character displacement between the sexes. Further, the relationship between an aquatic life history and sexual dimorphism (Figure 4) suggests that niche divergence associated with ecological speciation can affect the opportunity for the evolution of ecological sexual dimorphism, indicating that speciation and SA selection can interact to influence phenotypic diversification during adaptive radiation. Thus our data suggest that ecological sexual dimorphism is not only evolving together with ecological speciation, but also may be playing a key role in adaptation to aquatic life in *Notophthalmus*.

Although theoretical models of sympatric speciation, under some conditions, can predict an outcome of orthogonal divergence between the sexes and species (Cooper et al., 2011) similar to our results, speciation in *Notophthalmus* has likely involved a large allopatric component despite evidence of gene flow between nascent species (Gabor & Nice, 2004; Takahashi et al., 2014; although *N. perstriatus* is sympatric with and the sister species of *N. viridescens*, too little is known about the historic range of *N. perstriatus* to make any confident claims on the geography of speciation). Current and past work in *Notophthalmus* thus suggests the following scenario for the evolution sexual dimorphism during probable allopatric ecological speciation. First, ecological divergence during speciation appears to reflect adaptive changes in the life history in response to geographic variation in pond hydroperiod and terrestrial habitat quality; body size divergence during speciation likely reflects divergent size optima associated with life histories characterized by complete ontogenetic niche shifts vs a more aquatic life cycle (Takahashi & Parris, 2008; Takahashi, Takahashi, & Parris, 2010; Takahashi et al., 2011). Second, the correspondence between within‐species estimates of SA disruptive natural selection and divergence in sexual dimorphism across species indicates that sexual dimorphism in head shape in *Notophthalmus* may in part represent the outcome of within‐pond resource competition. Taken together, our work suggests that the evolution of sexual dimorphism could play a key role in ecological speciation in *Notophthalmus* by resolving sexual conflict arising from SA natural selection and reducing intraspecific resource competition associated with life in aquatic environments. This interpretation is consistent with the idea that the evolution of sexual dimorphism may play an important role in ecological speciation by facilitating adaption and thus population persistence.

Our results and interpretation of morphological evolution in *Notophthalmus* are also broadly consistent with recent ideas that suggest sexual antagonism and its resolution may go hand in hand with local adaptation (Connallon, 2015), environmental change (Connallon & Hall, 2016), and adaptive radiation (De Lisle & Rowe, 2015b). For example, our past work suggests a role for resource competition in generating SA natural selection, yet it is unlikely that sexual dimorphism in morphology and habitat use in *Notophthalmus* is a sole outcome of resource competition. Females of all species oviposit in the benthos, often for extended periods of the year, and appear also to use the benthos as a refuge from male premating struggles (Grayson et al., 2012), and so it is likely that divergent sex roles and resource competition between the sexes both contribute to generating sexual antagonism and consequently the evolution of sexual dimorphism. This also suggests that it could perhaps be unlikely to expect disruptive selection within breeding ponds to lead to sympatric speciation, if this disruptive selection is also related to the sex roles (i.e., ecological opportunity is sex‐specific; De Lisle & Rowe, 2015b). Such a scenario of a constraining effect of sexual dimorphism on speciation would be expected to manifest when disruptive selection, ecological sexual dimorphism, and divergence between nascent species all align. Our data are inconsistent with this. If SA natural selection generated by resource competition between the sexes typically aligns with directions in phenotype space where the sexes have already begun to diverge due to selection directly related to the sex roles, as is theoretically likely to be the case (De Lisle & Rowe, 2015a), then it may be generally unlikely to expect a constraining effect of character displacement between the sexes on ecological speciation. This suggestion begs more empirical tests of competition's role in the evolution of sexual dimorphism in other study systems.

Subspecies of *N. viridescens* represent ecologically distinct groups that have arisen rapidly in the last 10,000 years via niche divergence during range expansion from glacial refugia (Takahashi et al., 2014), yet little is known about where these subspecies lie on the speciation continuum. Although there is some evidence of assortative mating by body size, consistent with our finding of a large size component to among‐species and subspecies divergence, prezygotic reproductive isolation appears to be incomplete between some subspecies (Takahashi et al., 2010). Although lack of complete speciation in this clade does not effect the interpretation of our data, as subspecies do represent ecologically‐ and phenotypically diverged entities (Takahashi et al., 2014), future work examining the extent of pre‐ and postzygotic reproductive isolation in *N. viridescens*, experimental assessment of divergent selection and variation in sexual dimorphism across subspecies ranges would be informative.

We have interpreted our results predicated on the possibility that resource competition may be a perennial driver of disruptive selection for aquatic adult *Notophthalmus*. Although we have demonstrated disruptive selection on the same morphological traits examined here in three independent treatments/experiments in one subspecies of *N. *viridescens (De Lisle & Rowe, 2015a), we have no estimates of selection from other taxa in the genus. However, two pieces of evidence support the predication that disruptive natural selection may be general across the genus. First, all subspecies and species of *Notophthalmus* are (partially) aquatic predators that share the same feeding apparatus and inhabit standing water bodies (Petranka, 1998) that would be expected to share similar distributions of aquatic prey. Second, theory suggests that under conditions of a relatively constant resource distribution, frequency‐dependent competition creates stable fitness minima that maintains pervasive disruptive selection (Abrams, Matsuda, & Haranda, 1993; Rueffler et al., 2006).

Our work also adds to a small but growing number of empirical studies that have related properties of the adaptive landscape, as inferred from within‐population selective surfaces, to macroevolutionary patterns of phenotypic divergence (Chenoweth, Rundle, & Blows, 2010; Hohenlohe & Arnold, 2008; Punzalan & Rowe, 2016). These studies provide the exciting suggestion that in some cases microevolutionary quantitative genetics can deepen our understanding of macroevolutionary dynamics. Unlike past studies, which have focused on adaptive landscapes characterized by multivariate stabilizing selection, we have shown a role for disruptive selection in predicting variation across a clade. Given the strong theoretical expectations for pervasive disruptive selection under appropriate ecological conditions, it may be the case that such forms of selection lead to conserved patterns of divergence in other lineages.

Despite theoretical interest, ecological causes of dimorphism and corresponding effects on adaptive radiation have received limited empirical attention. This is especially true when considered in comparison with sexual selection (Panhuis, Butlin, Zuk, & Tregenza, 2001; Schluter, 2000; Wagner, Harmon, & Seehausen, 2012), an alternative (but not mutually exclusive) cause of phenotypic divergence between the sexes, which is viewed as an important component of adaptive radiation by creating and maintaining reproductive barriers between nascent species. In contrast, ecological sexual dimorphism is typically viewed as a constraint on adaptive radiation. Yet empirical examples of a constraining effect of sexual dimorphism on adaptive radiation are limited and lack explicit evidence for character displacement between the sexes (Butler et al., 2007; Schoener, 1977). We have shown that sexual dimorphism driven by ecological character displacement, and ecological speciation, can occur together during adaptive radiation in different combinations of traits. Thus, speciation and the evolution of ecological sexual dimorphism need not be strange bedfellows.

## Conflict of interest

None declared.
