# Supplementary material for: Disruptive natural selection predicts divergence between the sexes during adaptive radiation
Source: Ecol Evol. 2017 Apr 11;7(10):3590–601. doi: 10.1002/ece3.2868 (PMC5433998; doi:10.1002/ece3.2868)
Supplement: Supplementary file 1 [file ECE3-7-3590-s001.docx]

**Appendix**

*Analysis of Brownian motion analogues of* **D** *and* **S**

We estimated Brownian motion rate analogues of among-taxa covariance matrices as using the maximum likelihood estimator

|  | $\mathbf{R}=\frac{\left( \mathbf{Y}-\mathbf{1}\boldsymbol{\mu}^{\mathbf{T}} \right)^{T}\mathbf{C}^{\mathbf{-1}}\left( \mathbf{Y}-\mathbf{1}\boldsymbol{\mu}^{\mathbf{T}} \right)}{n}$ |  |
| --- | --- | --- |

where **R** is the Brownian motion rate matrix, **Y** is the *n* x *m* matrix of *m* trait values (either phenotypic means for **D** or canonical coefficients for **S**) from *n* taxa, $\boldsymbol{\mu}$ is the vector of trait phylogenetic means, **1** is a *n* x 1 vector of ones, and **C** is the phylogenetic covariance matrix specifying expected correlations among taxa under Brownian motion evolution along a phylogeny (Revell and Harmon 2008). We obtained a phylogeny of *Notophthalmus* from a recent amphibian phylogeny (Pyron and Wiens 2011) dated by penalized maximum likelihood (De Lisle and Rowe 2015b). We treated subspecies of *N. viridescens* as a polytomy with a crown age of 60 TYA (Takahashi et al. 2014). Our phylogeny is presented in Fig S1. We estimated Brownian motion rate matrices according to the above estimator using phytools (Revell 2012) in R. Estimates and spectral decompositions of these matrices are presented in Table S3. We found a strong correlation between the Brownian motion **s_max_** and **γ_max_** (*r_v_* = 0.78). Conversely, we found a weak association between Brownian motion **d_max_** and **γ_max_** (*r_v_* = 0.08).

**Appendix References**

De Lisle, S. P. and L. Rowe (2015). Independent evolution of the sexes promotes amphibian

diversification. Proceedings of the Royal Society B: Biological Sciences 282: 20142213.

Pyron, R. A. and J. J. Wiens (2011). A large-scale phylogeny of Amphibia including over 2,800

species, and a revised classification of extant frogs, salamanders, and caecilians.

Molecular Phylogenetics and Evolution 61(543-583).

Revell, L. J. (2012). Phytools: an R package for phylogenetic comparative biology (and other

things). Methods in Ecology and Evolution 3(3): 217-223.

Revell, L. J. and L. J. Harmon (2008). Testing quantitative genetic hypotheses about the

evolutionary rate matrix for continous characters. Evolutionary Ecology Research 10: 311-331.

Takahashi, M. K., et al. (2014). A stable niche assumption-free test of ecological divergence.

Molecular Phylogenetics and Evolution 76: 211-226.

**Figure S1.** Phylogeny of *Notophthalmus* used for estimation of Brownian motion rate analogues of **S** and **D** matrices. See Appendix text for details.

| **Table S1**. Phenotypic means (with standard deviation) for all traits measured from all species and subspecies of *Notophthalmus* | | | | | | | | | | |
| --- | --- | --- | --- | --- | --- | --- | --- | --- | --- | --- |
|  |  |  |  |  |  |  |  |  |  |  |
| Species or Subspecies |  |  |  |  |  |  | Head Depth |  | Jaw Length |  |
|  | Sex | N | SVL |  | Gape |  |  |  |  |  |
| *N. v. viridescens* | F | 55 | 43.81 | 3.69 | 7.62 | 0.63 | 4.37 | 0.36 | 6.37 | 0.70 |
|  | M | 67 | 43.77 | 3.14 | 7.30 | 0.52 | 4.44 | 0.32 | 6.52 | 0.57 |
| *N. v. dorsalis* | F | 27 | 38.40 | 3.81 | 6.87 | 0.54 | 4.11 | 0.33 | 5.90 | 0.55 |
|  | M | 28 | 36.39 | 3.02 | 6.34 | 0.50 | 3.96 | 0.41 | 5.95 | 0.41 |
| *N. v. louisianensis* | F | 87 | 39.32 | 5.03 | 7.13 | 0.84 | 4.00 | 0.46 | 5.94 | 0.69 |
|  | M | 74 | 36.72 | 3.17 | 6.53 | 0.54 | 3.93 | 0.33 | 6.07 | 0.50 |
| *N. v. piaropicola* | F | 26 | 39.87 | 4.19 | 7.21 | 0.73 | 3.99 | 0.34 | 5.89 | 0.44 |
|  | M | 26 | 34.60 | 2.21 | 6.25 | 0.49 | 3.76 | 0.26 | 5.73 | 0.35 |
| *N. perstriatus* | F | 16 | 31.23 | 2.86 | 5.45 | 0.62 | 3.37 | 0.21 | 4.97 | 0.53 |
|  | M | 27 | 28.58 | 2.33 | 4.97 | 0.44 | 3.24 | 0.27 | 5.06 | 0.34 |
| *N. meridionalis* | F | 20 | 44.67 | 7.16 | 7.98 | 0.92 | 4.08 | 0.57 | 6.60 | 0.78 |
|  | M | 24 | 41.94 | 5.62 | 7.59 | 0.77 | 3.94 | 0.53 | 6.42 | 0.49 |
|  |  |  |  |  |  |  |  |  |  |  |

| **Table S2.** Canonical discriminant function analyses of male and female morphology across the genus *Notophthalmus* | | | | | | | | |
| --- | --- | --- | --- | --- | --- | --- | --- | --- |
| ***Notophthalmus v. viridescens*** | | | |  | ***Notophthalmus v. dorsalis*** | | | |
|  | Discriminant Function | | Canonical Coefficients |  |  | Discriminant Function | | Canonical Coefficients |
| Variable | Male | Female |  |  | Variable | Male | Female |  |
| Constant | -105.70 | -106.63 | - |  | Constant | -101.98 | -107.27 | - |
| SVL | 0.58 | 0.57 | 0.01 |  | SVL | -0.60 | -0.57 | 0.07 |
| Gape | 8.49 | 11.29 | -1.47 |  | Gape | 6.38 | 11.00 | 1.74 |
| Head Depth | 19.21 | 16.04 | 0.96 |  | Head Depth | 19.28 | 17.39 | -0.46 |
| Jaw Length | 5.95 | 5.04 | 0.51 |  | Jaw Length | 18.34 | 15.18 | -0.97 |
| Error Rate | 0.284 | 0.236 | - |  | Error Rate | 0.178 | 0.185 | - |
| N = 122; Canonical correlation = 0.49; SE = 0.069; P <0.0001 | | | |  | N = 55; Canonical correlation = 0.618; SE = 0.084; P <0.0001 | | | |
|  |  |  |  |  |  |  |  |  |
| ***Notophthalmus v. louisianensis*** | | | |  | ***Notophthalmus v. piaropicola*** | | | |
|  | Discriminant Function | | Canonical Coefficients |  |  | Discriminant Function | | Canonical Coefficients |
| Variable | Male | Female |  |  | Variable | Male | Female |  |
| Constant | -54.68 | -56.04 | - |  | Constant | -114.36 | -128.15 | - |
| SVL | -0.55 | -0.24 | -0.80 |  | SVL | 0.68 | 1.27 | 1.23 |
| Gape | 2.63 | 6.08 | -1.56 |  | Gape | 1.26 | 4.00 | 1.06 |
| Head Depth | 13.57 | 9.40 | 0.99 |  | Head Depth | 10.93 | 3.61 | -1.17 |
| Jaw Length | 9.70 | 6.80 | 1.04 |  | Jaw Length | 27.24 | 27.55 | 0.06 |
| Error Rate | 0.202 | 0.195 | - |  | Error Rate | 0.039 | 0.192 | - |
| N = 161; Canonical correlation = 0.654; SE = 0.045; P <0.0001 | | | |  | N = 52; Canonical correlation = 0.719; SE = 0.068; P <0.0001 | | | |
|  |  |  |  |  |  |  |  |  |
| ***Notophthalmus perstriatus*** | | | |  | ***Notophthalmus meridionalis*** | | | |
|  | Discriminant Function | | Canonical Coefficients |  |  | Discriminant Function | | Canonical Coefficients |
| Variable | Male | Female |  |  | Variable | Male | Female |  |
| Constant | -139.35 | -136.42 | - |  | Constant | -69.15 | -73.33 | - |
| SVL | 3.00 | 3.27 | -0.42 |  | SVL | -2.37 | -2.20 | -1.42 |
| Gape | -32.16 | -26.76 | -1.61 |  | Gape | 20.16 | 22.02 | -2.05 |
| Head Depth | 50.30 | 47.40 | 0.39 |  | Head Depth | -1.46 | -5.08 | 2.58 |
| Jaw Length | 37.46 | 31.52 | 1.31 |  | Jaw Length | 14.10 | 13.62 | 0.40 |
| Error Rate | 0.111 | 0.125 | - |  | Error Rate | 0.333 | 0.250 | - |
| N = 43; Canonical correlation = 0.681; SE = 0.083; P <0.0001 | | | |  | N = 44; Canonical correlation = 0.367; SE = 0.132; P = 0.216 | | | |

| **Table S3.** ML estimates and spectral decompositions of Brownian motion analogues of **S** and **D** matrices. | | | | | | | | | | |
| --- | --- | --- | --- | --- | --- | --- | --- | --- | --- | --- |
| **S** | | | | |  | eigenvalues | eigenvectors | | | |
| Trait | SVL | Gape | Head Depth | Jaw Length |  |  | SVL | Gape | Head Depth | Jaw Length |
| SVL | 71.55 | 91.87 | -78.31 | -30.67 |  | 491.091 | 0.280 | 0.769 | -0.464 | -0.338 |
| Gape | - | 296.52 | -169.30 | -134.32 |  | 57.984 | 0.713 | -0.298 | -0.414 | 0.481 |
| Head Depth | - | - | 117.58 | 62.60 |  | 10.746 | 0.579 | -0.248 | 0.415 | -0.656 |
| Jaw Length | - | - | - | 74.18 |  | 0.012 | 0.278 | 0.507 | 0.664 | 0.474 |
|  |  |  |  |  |  |  |  |  |  |  |
| **D** | | | | |  | eigenvalues | eigenvectors | | | |
| Trait | SVL | Gape | Head Depth | Jaw Length |  |  | SVL | Gape | Head Depth | Jaw Length |
| SVL | 30.04 | 21.47 | 25.93 | 22.18 |  | 84.637 | 0.594 | 0.421 | 0.522 | 0.444 |
| Gape | - | 16.08 | 17.51 | 15.70 |  | 2.308 | 0.203 | 0.679 | -0.699 | -0.093 |
| Head Depth | - | - | 24.20 | 19.73 |  | 0.268 | -0.520 | 0.082 | -0.182 | 0.831 |
| Jaw Length | - | - | - | 16.91 |  | 0.025 | -0.579 | 0.596 | 0.453 | -0.322 |
|  |  |  |  |  |  |  |  |  |  |  |
